# Effect of human immunodeficiency virus on blood-brain barrier integrity and function: an update

**DOI:** 10.3389/fncel.2015.00212

**Published:** 2015-06-10

**Authors:** Venkata Subba Rao Atluri, Melissa Hidalgo, Thangavel Samikkannu, Kesava Rao Venkata Kurapati, Rahul Dev Jayant, Vidya Sagar, Madhavan P. N. Nair

**Affiliations:** Department of Immunology, Institute of NeuroImmune Pharmacology, Herbert Wertheim College of Medicine, Florida International UniversityMiami, FL, USA

**Keywords:** HIV, gp120, Tat, Nef, Vpr, blood-brain barrier, neurocognitive disorders

## Abstract

The blood-brain barrier (BBB) is a diffusion barrier that has an important role in maintaining a precisely regulated microenvironment protecting the neural tissue from infectious agents and toxins in the circulating system. Compromised BBB integrity plays a major role in the pathogenesis of retroviral associated neurological diseases. *Human Immunodeficiency Virus* (HIV) infection in the Central Nervous System (CNS) is an early event even before the serodiagnosis for HIV positivity or the initiation of antiretroviral therapy (ART), resulting in neurological complications in many of the infected patients. Macrophages, microglia and astrocytes (in low levels) are the most productively/latently infected cell types within the CNS. In this brief review, we have discussed about the effect of HIV infection and viral proteins on the integrity and function of BBB, which may contribute to the progression of HIV associated neurocognitive disorders.

## Introduction

**Figure 1:**
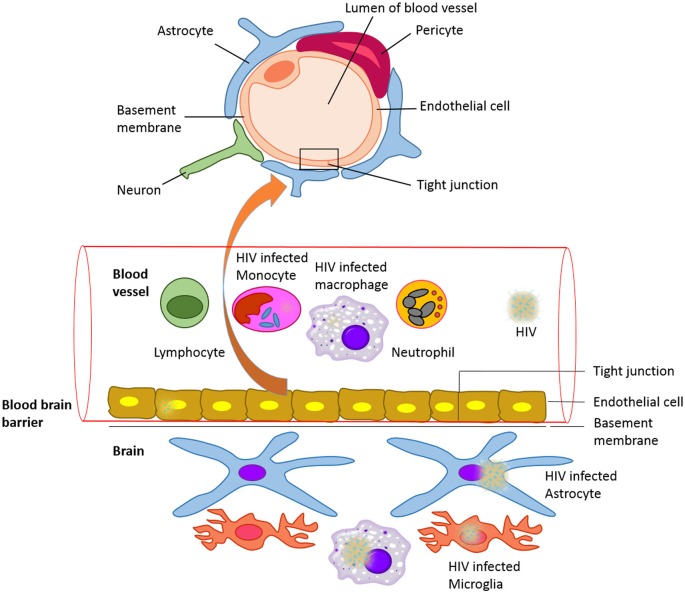
**The blood-brain barrier (BBB) with HIV infected cells: Schematic representation of structure of the blood brain barrier and HIV infected cells in blood and CNS**.

The brain functions within a well-controlled environment that is quite distinct from other parts of the body. Any changes in the environment of the brain result in reversible or irreversible and fatal or non-fatal conditions. The blood-brain barrier (BBB) is a diffusion barrier essential for the normal function of the central nervous system (CNS). Ballabh et al. (2004) and Weiss et al. (2009) have overviewed the structure and functional role of the BBB in brain homeostasis. The BBB is a protective, selectively permeable barrier that protects the brain from foreign substances (infectious agents and neurotoxic substances) in blood that might damage the brain. It also protects the brain from many circulating hormones and neurotransmitters in the peripheral system of the body. Apart from providing a secure environment for neural network in the brain, it acts as a regulator of energy metabolites, specific ion channels and transporters. For example, water, glucose, amino acids and some lipid soluble molecules that are essential for neuronal growth and function will pass through the BBB by passive diffusion and selective transport mechanisms, while preventing the entry of lipophilic and potential neurotoxic substances. The BBB endothelial cells are different from the ones in the rest of the body since they lack fenestrations, have more broad tight junctions (TJs), and a scant pinocytic vesicular transport. In addition, the BBB is made of the capillary basement membrane (BM), pericytes (PCs) embedded within the BM and astrocyte end-feet enclosing the vessels (Figure 1).

Infections of the CNS are very common and cause high morbidity and mortality in children as well as in immunocompromised persons. The most common organisms involved in CNS infections are *Streptococcus pneumoniae*, *Hemophilus influenzae* type B, *Neisseria meningitides, Listeria monocytogenes*, and *Human Immunodeficiency Virus* (HIV) type-1. HIV-1 exhibits extensive genetic variation worldwide and is categorized into three groups (M, O, and N) and genetically into nine different subtypes (A–K). Of these, clades B and C represent the majority (>86%) of circulating HIV-1 variants (Osmanov et al., 2002). While HIV-1 clade B is predominant in North America, Western Europe, and Australia; clade C is common in Southern and East Africa, India and Nepal and is responsible for around half of all HIV infections. HIV is a neurotropic virus causing inflammatory and neurotoxic host responses. Severe neurological disorders caused by HIV are collectively known as HIV-associated neurocognitive disorders (HAND). HAND is characterized by the development of cognitive, motor and/or behavioral impairments and consists of several clinical forms ranging from asymptomatic neurocognitive impairment (ANI), minor neurocognitive disorder (MND) to the most severe HIV-associated dementia (HAD) (McArthur and Brew, 2010). HIV-encephalitis (HIVE) is the main cause of HAND and the most common neurologic disorder of the brain in HIV-1 infection. Disruption of the BBB caused by HIV-1 plays an important role in AIDS neuropathogenesis (Persidsky and Gendelman, 2003; Toborek et al., 2005), but the exact mechanisms of HIV entry into the brain across the BBB remain unclear. Numerous studies have used animal models as well as *in vitro* experimental studies to understand the mechanisms of HIV-1 introduction into the CNS through the BBB (González-Scarano and Martín-García, 2005).

The commonly recognized model, with the most valid evidence, is the “Trojan Horse hypothesis” (Peluso et al., 1985). Based on this model, HIV-1 and other lentiviruses cross the CNS as a passenger in cells trafficking to the brain. HIV-1 infects several CD4+ (cluster of differentiation 4) cells, such as T cells and monocytes. These cells circulate in the blood, cross the BBB and propagate the infection within the CNS (Figure 1). It has also been shown that macrophage tropism is a prerequisite for the neurotropism of HIV. This study was done using molecularly cloned *simian immunodeficiency virus* in macaques (SIVmac) 239, which is a lymphocyte virus rather than macrophagetropic. This study showed that after intracerebral (i.c.) inoculations, the infection propagated in lymphoid organs and bone marrow, and did not infect the brain directly. This resulted in severe neurological disease and classical neuropathological injuries (Sharma et al., 1992). Other proposed mechanisms include direct infection of cells that comprise the BBB, endothelial cells and astrocytes, and transfer via these cells from the periphery to the CNS (Mankowski et al., 1994; Argyris et al., 2003; Bobardt et al., 2004) or direct extracellular movement of the virus across a disrupted BBB. Even though these mechanisms are important to take in consideration, they are less well characterized, and are possible minor contributors to CNS infection. HIV replicates in brain microglia as well as macrophages, producing inflammatory and neurotoxic host responses (Anthony et al., 2005). It has been demonstrated that HIV-1 infection of human brain PCs and disruption of BBB integrity might also contribute to the progression of HIV-1 induced CNS damage (Nakagawa et al., 2012). HIV may cause cognitive, behavioral, and motor difficulties in 40–60% of infected individuals even in the current antiretroviral therapy (ART) era (Anthony et al., 2005). These difficulties may range in severity from very mild to severe and disabling.

Since the BBB does not allow easy transfer of any foreign molecules, treatments of these infections become more difficult (Guduru et al., 2013; Pilakka-Kanthikeel et al., 2013; Ding et al., 2014; Jayant et al., 2015). The BBB integrity is disrupted throughout the pathogenesis of neuroAIDS by mechanisms that comprise higher transmigration of HIV-infected monocytes, decline in BBB TJ protein expression, and apoptosis (Dallasta et al., 1999; Eugenin et al., 2006). The aim of this review is to update the effect of HIV infection and role of different HIV proteins in BBB integrity and functions.

## HIV Infection of CNS Cells and Effect on BBB Permeability

The BBB composition consists of highly specialized microvascular endothelial cells that are found on the basal lamina. Astrocyte end feet interaction with the basal lamina and their proximity to endothelial cells and accessory cells including microglia, perivascular macrophages and PCs has been observed (Abbott, 2002; Ballabh et al., 2004). The processes of astrocytes end feet surround the brain capillaries, giving important factors for the maintenance and development of the BBB (Goldstein, 1988; Risau and Wolburg, 1990; Risau, 1991; Hayashi et al., 1997). These astrocytes end feet aid in the regulation of the BBB physiology using soluble factors (Rubin and Staddon, 1999; Hawkins and Davis, 2005; Iadecola and Nedergaard, 2007), direct intercellular communication through gap junction (GJ) channels and by connexin/pannexin hemichannels found on cells surface, in which the cytoplasm and the extracellular environment join together. GJs are a combination of channels that allow the interchange of particles up to 1.2 kDa among the cytoplasm of nearby cells (Saez et al., 2003). Every channel is composed of the cutting of two hemichannels, which are found in opposing cell membranes; each hemichannel is a group of six connexins (Cxs). GJ connection decreases during pathological settings, such as inflammation and viral infections (Danave et al., 1994; Faccini et al., 1996; Rouach et al., 2002; Kielian and Esen, 2004; Knabb et al., 2007). Due to the expression of connexin43 (Cx43) and functional GJ communication among astrocytes, toxic signals produced in the HIV infected astrocytes extent to the nearby uninfected astrocytes (Eugenin and Berman, 2007). Cell-to-cell interaction has been shown to be an effective transmission pathway of HIV from T lymphocytes to astrocytes. This was observed through a dynamic binding process of filopodial extensions from either cell type, developing virological synapses. These interactions could be inhibited by anti-C-X-C chemokine receptor type 4 (CXCR4) antibody and its antagonist (Ma et al., 2015).

In the brain, perivascular macrophages and microglia are the productively infected cell types by HIV *in vivo* (Wiley et al., 1986; Cosenza et al., 2002). Studies have shown that SIV-infected macrophages lower electrical resistance of the endothelial cell monolayer after adding them to the parenchymal face of an inverted BBB model (Sansing et al., 2012). Using an *in vitro* BBB model, HIV infection of leukocytes reported to increase their transmigration across the BBB in response to the chemokine ligand 2 (CCL2) as a result of increased permeability, reduced TJ proteins such as zonula occludens-1 (ZO-1), claudin-1, and occludin in the BBB cells, and up-regulation of matrix metalloproteinases (MMP)-2 and MMP9 (Eugenin et al., 2006). Nevertheless, astrocytes have also been reported to get infected by HIV in low levels (Wiley et al., 1986; Conant et al., 1994; Nuovo et al., 1994; Tornatore et al., 1994; Bagasra et al., 1996; Takahashi et al., 1996; Ohagen et al., 1999; Eugenin and Berman, 2007; Churchill et al., 2009; Atluri et al., 2013). C-C chemokine receptor type 5 (CCR5) and CXCR4 are functional HIV co-receptors observed in astrocytes, which assist in infection (Klein et al., 1999). Even though a small amount of infected astrocytes were observed *in vitro* (4.7 ± 2.8% of the total astrocytes in the culture) as well as a low HIV production, GJ channels intensify and extent toxic signals to the adjacent cells in the culture that were not infected (Eugenin and Berman, 2007). It has been observed that this small population of infected astrocytes, with significant viral production, is able to cause intense modifications in the physiology of the BBB *in vitro* as well as *in vivo* when SIV-infected macaques brain sections were used (Eugenin et al., 2011). In addition, HIV infection of human astrocytes was demonstrated to induce increased expression of dickkopf-1 protein (DKK1; a soluble inhibitor of Wnt signaling) by a Cx43 hemichannel HC-dependent mechanism, which is associated with the brain pathogenesis found in HIVE patients. Even in the absence of HIV infection, co-cultured primary neurons and astrocytes in the presence of increasing concentrations of DKK1 resulted in neuronal damage (Orellana et al., 2014). One of the major notable pathological features in neuroAIDS has been reported to be the disruption of the BBB (Dallasta et al., 1999; Luabeya et al., 2000; Eugenin et al., 2006). To see the effect of HIV infected astrocytes on BBB permeability, a BBB was constructed using infected/uninfected astrocytes and Evans Blue (EB) conjugated albumin permeability test was used. In the BBB with HIV infected astrocytes, high permeability, endothelial apoptosis, erroneous astrocyte end processes and abnormal interactions with the endothelium were reported (Eugenin et al., 2011).

## Effect of HIV Proteins on BBB Permeability and Function

It is well known that HIV-1 genome encodes *Gag, Pol*, and *Env* polyproteins which are subsequently proteolyzed into individual proteins. The four *Gag* proteins, matrix, capsid, nucleocapsid and p6 proteins and the two *Env* proteins, surface gp120, transmembrane gp41 are structural proteins that makeup the core and outer membrane of the virion. The three *Pol* proteins protease, reverse transcriptase, and integrase are encapsulated within the particle and play a major role in viral replication (Frankel and Young, 1998).

## Effect of HIV gp120 Protein on BBB Permeability

Gp120 is a potent neurotoxin, causing neurotoxicity even in pico molar concentration and has been detected in the serum of HIV-infected patients (Ellaurie et al., 1990). Viral entry into the host cell is initiated by binding of the gp120 located on the viral membrane surface to a specific cell specific receptor (especially CD4) (Frankel and Young, 1998). HIV-1 co-receptors CCR5 and CXCR4 are expressed by human brain microvascular endothelial cells (hBMVECs). When these cells were exposed to HIV gp120 protein, derived from HIV infected lymphocytes or macrophage-tropic viruses, endothelial monolayer permeability and monocyte migration in *in vitro* models were increased (Kanmogne et al., 2007). In an *in vitro* setup of brain endothelial cells obtained from gp120 transgenic mice and non-transgenic mice, increased permeability of endothelial cell layer exposed to the serum containing gp120 was reported and the permeability was restored by using the anti-gp120 antibody demonstrating the effect of circulating gp120 on BBB permeability (Cioni and Annunziata, 2002). The gp120 also produced down-regulation of TJ proteins, such as ZO-1, ZO-2, and occludin, functions in cross-linking which led to the increased permeability of the endothelial mono layer (Kanmogne et al., 2005). It has been shown that TJ disturbance among hBMVECs is mediated by activating the focal adhesion kinase (FAK) through phosphorylation at tyrosine (TYR)-397. Nevertheless, inhibiting FAK activation was enough to avoid disturbance of TJ (Ivey et al., 2009).

It has also been reported that gp-120 proteins increased the expression of MMPs 2 and 9 and subsequently Claudin-5 and laminin, which are key targets of these MMPs were reduced. In addition, even acute exposure of gp120 intensifies lipid peroxidation in neurons as well as in the vascular endothelium. Further, administration of antioxidants could be able to protect the BBB from the gp120-induced BBB damage (Louboutin et al., 2010). Moreover, studies have shown that lymphatic hyperpermeability was induced by gp120 through the functional disruption of Robo4, a novel regulator of endothelial permeability. HIV-1 gp120 caused integrin α5β1 phosphorylation and fibronectin expression, which led to their association with Robo4 through its fibronectin type III domains (Zhang et al., 2012). On the other hand, when an active N-terminus portion of Slit2, a Robo4 agonist, was used for pretreatment, lymphatic endothelial cells demonstrated to be protected against HIV-1 gp120-induced hyperpermeability. This happened through the inhibition of c-Src kinase activation (Zhang et al., 2012).

CCR5 antibodies and inhibitors of myosin light chain kinase or protein kinase C (PKC) were able to block and reduce the permeability of BBB *in vitro* as well as monocyte migration due to the exposure of gp120. In addition, CCR5 antibody and CXCR4 antagonist prevented and partially blocked the gp120 induced release of intracellular calcium, respectively. Some junctional proteins, such as claudin-1 and claudin-5 did not show any change or effect. Specific PKC inhibitors, acting at the calcium release and adenosine triphosphate (ATP)-binding site, prevented intensification in BBB permeability and blocked gp120-induced PKC (PKC activation). Additionally, the integrity of the BBB was restored after the removal of gp120 (Kanmogne et al., 2007). Studies have also shown that hBMVECs treated with proteasome inhibitor, lactacystin, reduced the deprivation of ZO-1 and ZO-1. Up-regulation of a scaffold protein, 14-3-3τ, which is a negative regulator of TJ proteins has also been reported in the presence of gp120. These results indicate that TJ proteins were directed by gp120 for deprivation through the proteasome, portraying a novel molecular mechanism for the BBB breakdown by gp120 (Nakamuta et al., 2008).

Furthermore, when immortalized endothelial cell line from rat brain capillaries (RBE4) were treated with gp120 and Tat, levels of glutathione peroxidase (GPx), intracellular reduced glutathione (GSH), and glutathione reductase (GR) content were decreased while levels of malondialdehyde (MDA) were increased. Reduced glutathione and oxidized glutathione (GSH/GSSG) ratio was taken in consideration as an oxidative stress indicator, in which the ratio was reduced in both gp120 and Tat exposed RBE4 cells (Price et al., 2005). These outcomes demonstrated that cellular expression of gp120 increased MMPs and reduced vascular TJ proteins, leading to a disturbance of the BBB permeability. This confirms that HIV gp120 induces oxidative damage and production of reactive oxygen species (ROS), which lead to the alteration of the BBB integrity (Louboutin et al., 2010). These changes increase the trafficking of toxic humoral factors and HIV infected cells into the CNS, which contribute to the pathogenesis of severe forms of HIV associated neurocognitive disorders i.e., HAD (Kanmogne et al., 2005). In addition, it was demonstrated that, up-regulation of cannabinoid receptor 2 (CB2R) levels in brain endothelium during neuroinflammation is able to increase the quantity of TJ protein found in membrane fractions. Further, in lipopolysaccharide administered *in vivo* mouse model, the role of CB2R in attenuation of inflammatory response and in preventing BBB leakiness was confirmed by using CB2R agonists (Ramirez et al., 2012). In an *in vivo* experimental male Wistar rat model, up regulation of IL-1β and inducible nitric oxide synthase (iNOS) transcripts were also observed in the presence of gp120. However, anti-inflammatory compounds, such as minocycline, chloroquine and simvastatin, are able to decrease neuroinflammation induced by gp120. This effect has been reported to be mediated by the interaction with the mitogen-activated protein kinase (MAPK) signaling pathway (Ashraf et al., 2014). In an *in vivo* rat caudate-putamen gp120 and EB intravenous injection study, gp120 induced extravasation of the EB (Louboutin and Strayer, 2012). This information reveals that HIV gp120 proteins affect the functional as well as molecular properties of the BBB.

## Effect of HIV Trans-Activator of Transcription (Tat) Protein on BBB Permeability

Despite the presence of different cellular transcription factors like nuclear factor kappa-light-chain-enhancer of activated B cells (NF-kB), specificity protein 1 (Sp1) and TATA-binding protein (TBP), which help in transcriptional initiation from the integrated provirus, Tat is required to enhance the processivity of transcribing polymerases. Tat is also able to increase the rate of transcription initiation and production of viral mRNAs more than 100 fold, consequently required for viral replication (Frankel and Young, 1998). HIV Tat has also shown to have effects on BBB integrity mediated by oxidative stress (Price et al., 2005; Pu et al., 2005). Although studies have shown that Tat affects TJ protein expression, detailed mechanisms have not been clearly described yet. HBMVECs that were exposed to HIV Tat revealed a decline in occludin mRNA and protein levels via RhoA/ROCK signaling pathway. However, p160-Rho-associated coiled kinase (ROCK) inhibitor Y-27632 and RhoA inhibitor C3 exoenzyme moderately diminished the effect (Xu et al., 2012).

Additionally, Rho signaling in HBMVECs was demonstrated to have a significant role in monocyte migration through the BBB and in TJ assembly. Rho activation negatively regulates the TJ assembly and studies have shown that monocyte migration was decreased by 84% when dominant-negative Rho K-transfected BMVECs were utilized in BBB models (Persidsky et al., 2006). Exposure to Tat results in Rho activation, TJ integrity disruption and enhanced monocyte migration across the BBB which has been shown during the HIV infection as well (Persidsky et al., 2006; Zhong et al., 2012). Activated monocytes demonstrated higher quantities of lysosomes, philopodia, vesicular Golgi complexes as well as augmented expressed levels of proinflammatory cytokines, such as IL-6, IL-10, and TNF-alpha which further affect the BBB integrity (Persidsky et al., 1997).

CCL2/MCP-1 reported to induce chemotaxis of human microglia, indicating that uninfected cells can be induced to migrate areas of active infection or BBB disruption where CCL2/MCP-1 is released in response to Tat (Eugenin et al., 2005). Furthermore, Tat induced the expression of MMP-9 and the concentration of soluble occludin in cells’ supernatants and the paracellular permeability of hBMVEC treated with Tat decreased due to RNA interference targeting MMP-9. These data explain how Tat disturbs the integrity of BBB by diminishing occludin production and cleaving occludin through MMP-9 (Xu et al., 2012). Moreover, studies in hCMEC/D3 cell line treated with Tat demonstrated a down regulation in ZO-1 total levels, but an up regulation of ZO-1 expression in the nuclei. Nevertheless, when the Rho cascade was inhibited by infecting *Adenovirus* expressing exoenzyme C3 transferase (C3 *Adenovirus*), ZO-1 up regulation in the nucei and CREB activation induced by Tat were prevented. The depletion of CREB by infecting cells with certain shRNA lentiviral particles, attenuated Tat induced nuclear targeting of ZO-1, Rho signaling and transendothelial migration of monocytic cells. The CREB level depletion also inhibited Tat induced hyperpermeability of endothelial and BBB (Zhong et al., 2012). Particularly, peroxisome proliferator-activated receptor (PPAR)α or PPARγ up-regulation attenuated HIV-mediated dysregulation of TJ proteins. These outcomes were also associated with the down regulation of MMP and protease activities. These studies clearly observed that HIV-induced down regulation of junctional adhesion molecule-A (JAM)-A and ZO-1 were restored by inhibiting MMP and proteasome activity by using the PPARγ agonists (15d-PGJ2 and troglitazone). This data indicated a novel mechanism of PPAR-induced defenses against HIV-induced disturbance of brain endothelial cells (Huang et al., 2009; Zhong et al., 2012). In the same way, Tat-induced synaptic loss and cell death were blocked by creatine and protected against Tat induced ATP depletion, proposing that it may be a convenient adjunctive therapy for HAND (Stevens et al., 2014).

In addition, *in vivo* experiments have shown that Tat crossed the BBB through a nonsaturable mechanism with a unidirectional inflow level of 0.490 μl/g/min. Approximately, 0.126% of an intravenous dose of Tat goes into each gram of brain (Banks et al., 2005). The uptake rate for Tat is analogous to the one at which several active proteins cross the BBB, such as IL-1 and TNF-alpha (Banks et al., 1991; Gutierrez et al., 1993). Tat is able to bind to different cell surface receptors, such as vascular endothelial cell growth factor receptor-2 on brain endothelial cells, low density lipoprotein receptor-related protein on brain neurons and the Fik-1KDR receptor on peripheral vascular endothelial cells. Definitely, it was observed that Tat crossed without disturbing the BBB, since radioactively labeled albumin that was injected simultaneously did not cross the BBB. These studies confirm that specific regions of the brain, such as the occipital cortex, hypothalamus and hippocampus are more permeable to Tat. Therefore, it was shown that Tat ended up crossing the BBB bidirectionally. Studies found that over 90% of the Tat taken up by the brain passed through the full width of the capillary wall (Banks et al., 1991, 2005; Albini et al., 1996; Liu et al., 2000; Khan et al., 2003). In addition, Tat may be crossing the BBB in the same way that HIV-1 gp120 does, through adsorptive endocytosis. The permeability observed in these studies, can give an insight and proposal of the mechanism involved by which the production of Tat on one side of the BBB could disturb immune or neural functions on the other side (Banks et al., 2005). Recently one study has shown that HIV-1 uses a uniform distribution of mannose-6 phosphate receptor (M6PR) to cross the BBB, using a transcytotic pathway (Dohgu et al., 2012).

HIV-1 Tat C protein has also been observed to increase intracellular ROS level and induce nicotinamide adenine dinucleotide phosphate-oxidase 2 (NOX2) and NOX4 expression levels in hBMVECs. An increased β-catenin and vascular endothelial-cadherin TYR phosphorylation by activation of Redox-sensitive kinase proline-rich tyrosine kinase 2 (PYK2), led to the disruption of junctional assembly. Phosphorylation of junctional proteins in hBMVECs caused disturbance in junctional complexes and endothelial permeability (Mishra and Singh, 2014).

## HIV-1 Negative Regulatory Factor (Nef) Protein on BBB Permeability

HIV-1 Nef protein has been shown to promote pathogenesis of HIV infection by facilitating the down-regulation of CD4 and through signal transduction interference by binding to cellular protein kinases and enhance the virus replication in the infected host (Frankel and Young, 1998; Abraham and Fackler, 2012). In animal models, MMP-9 activity was identified in CSF samples that were intracisternally injected with Nef. However, these results were not detected when heat-inactivated Nef, gp160, or gp120 were injected. This HIV-1 protein was observed to induce a rupturing of the BBB, which could be inhibited by pretreatment with MMP inhibitor batimastat. *In vitro* experiments showed that Nef induced MMP-9 activity in a lower level in isolated PBMCs and in the murine macrophage cell line RAW 264.7. However, these results were not observed in astroglial, neuronal or endothelial cell lines. These data point out that Nef is able to induce disturbance or disruption in the BBB, most likely via the induction of MMP (Sporer et al., 2000).

Nef has also demonstrated to play a major role in the depletion of T cells through the stimulation of apoptosis in bystander cells. Treatment with low levels of extracellular Nef concentrations, showed a moderate quantity of programmed cell death. On the other hand, when high levels of extracellular Nef concentrations were used, a significant induction of apoptosis was observed. Results also showed an up-regulation of proapoptotic genes, such as caspase-6, -8, -9, and -10 along with tumor necrosis factor receptor 12 (TNFR 12), tumor proteins p53 and p73, Death-associated protein 6 (DAXX), MAPK/extracellular signal-regulated kinase (ERK) 3, and MAPK 7 (Acheampong et al., 2005). Studies have also shown the suppression of B-cell help through HIV-1 infected T lymphocytes as well as a decrease in parenchymal lymphocyte motility, might contribute to the cell-associated virus spread of HIV infection in surrounding cells. These suggest not only that HIV-1 Nef induces apoptosis in hBMVECs, but that it may use different pathways in inducing AIDS pathogenesis (Stolp et al., 2012).

## HIV-1 Viral Protein R (Vpr) in BBB Permeability

**Figure 2:**
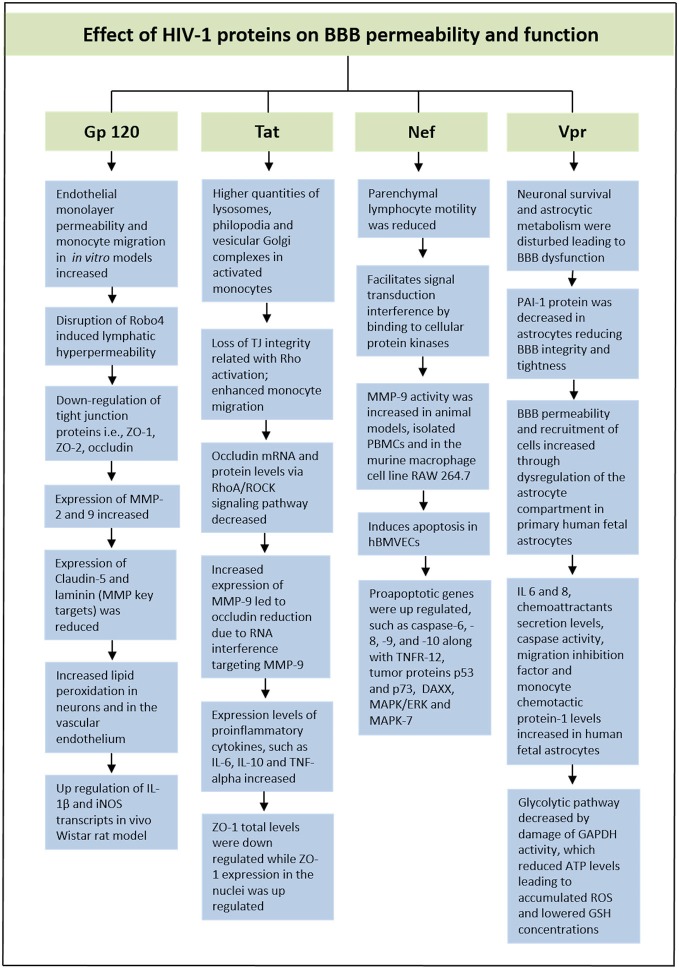
**Schematic representation showing effect of HIV proteins on BBB permeability and function**.

HIV-1 Viral Protein R (Vpr) is a very versatile or adaptable protein in HIV-1 life cycle that participate in cell cycle arrest at the G2 phase, transport of the pre-integration complex into the nucleus and activation of HIV-1 long-terminal repeat (Heinzinger et al., 1994; He et al., 1995; Vanitharani et al., 2001). According to some studies, decrease of plasminogen activator inhibitor-1 (PAI-1) protein in astrocytes exposed to Vpr could give harmful effects by reducing BBB integrity and tightness. A balanced production of this protein is vital for endothelial cells in the CNS. In exposure to primary human fetal astrocytes, extracellular Vpr has shown to contribute to an increased level in the permeability of the BBB along with an increased recruitment of cells through dysregulation of the astrocyte compartment, such as monocyte-macrophage cells infiltration into the CNS (Ferrucci et al., 2013). These HIV-1-induced symptoms affect disease progression as well as HIV pathogenesis. Furthermore, pro-inflammatory cytokines (IL 6 and 8), chemoattractants secretion levels, caspase activity, migration inhibition factor and MCP-1 levels were observed to increase in human fetal astrocytes. The glycolytic pathway was also observed to decrease by damage of Glyceraldehyde 3-phosphate dehydrogenase (GAPDH) activity, which led to reduced ATP levels (Ferrucci et al., 2013). The decrease in intracellular ATP will lead to an accumulation of ROS, which reduces GSH concentrations that affect a variety of genes in the Redox pathway. Additionally, SK-N-SH neuroblastoma cell line exposed to Vpr conditioned medium led to a reduction in GSH synthesis, resulting in apoptosis of the cells. Therefore, these data also showed how Vpr was able to ultimately disturb neuronal survival and affect astrocytic metabolism that lead to the BBB dysfunction (Ferrucci et al., 2013). In Figure 2, we have illustrated effect of HIV-1 proteins on BBB integrity and function.

## Targeting Active and Latent HIV in the Brain

The combination antiretroviral therapy (cART) and the available antiretroviral drugs are effective only in decreasing the viral load in the peripheral system but cannot eradicate the active or latent (reservoir) in the brain. The BBB is considered as a primary impediment barrier for the majority of drugs used in the treatment of HIV. Therefore, delivering antiretroviral drugs into the brain is still a big challenge to date. Based on the physico-chemical characteristics and the amount of drug concentration in the CNS, antiretroviral drugs were classified into low-CNS penetration, intermediate CNS penetration or high CNS penetration drugs (Letendre et al., 2008). Nanoparticles and nano-carrier based delivery of the anti-retroviral drugs across the BBB offer a great promise for neurotherapeutics (Vyas et al., 2006; Boisselier and Astruc, 2009; Nowacek and Gendelman, 2009; Mahajan et al., 2012).

## Nanotechnology Based Therapeutic Approach to Deliver Drugs Across BBB to Treat NeuroAIDS

Over the last few years, research studies have focused on exploring different nano-carriers systems to deliver anti-retro viral drugs to the brain (Wong et al., 2010; Sagar et al., 2014). Our lab is extensively studying the use of nano carrier system to deliver antiretroviral drugs and latency breaking agents into the brain. We have reported that multifunctional liposomal magnetic nanocarriers have an immense capacity for particle transmigration through the BBB and may possess a promising future in drug delivery to the brain (Saiyed et al., 2010; Pilakka-Kanthikeel et al., 2013; Ding et al., 2014). Recently, we have reported the use of sustain released magnetically guided layer-by-layer (LbL) assembled nanocarriers for the treatment of neuroAIDS. We have reported that the use of vorinostat and tenofovir in a single nanoformulation is helpful for activating latent viruses and killing them simultaneously (Atluri et al., 2014; Jayant et al., 2015).

## Conclusion

HIV-associated neurocognitive disorders are becoming a growing problem as the HIV-1-infected population ages. One of the pathological hallmarks of HAND and dementia is the BBB dysfunction that plays a critical role in this process. The BBB is more than ordinary hurdles to the passage of molecules and cells, and hence active players in brain homeostasis and its role in HIV-1 infection and HAND are still to be further recognized and investigated in the context of health and disease and in neurocognitive disorders. In this review, we emphasized that the communication between the periphery and the brain, through the brain barriers, is compromised during HIV-1 infection. Despite the well-accepted fact that the barriers are active participants in brain homeostasis, further studies are required to fully understand how the barriers function is altered during HIV-1 infection, possible compromise and its contribution to neurodegenerative diseases such as HAND and HIV associated dementia.

## Conflict of Interest Statement

The authors declare that the research was conducted in the absence of any commercial or financial relationships that could be construed as a potential conflict of interest.
